# Changes in soil microbial community structure during the transformation from native soil to alfalfa cultivation soil in the Kunlun Mountain sand area, Xinjiang, China

**DOI:** 10.3389/fmicb.2025.1702974

**Published:** 2025-12-17

**Authors:** Yongcheng Chen, Xiao Li, Rongzheng Huang, Fanfan Zhang, Chunhui Ma

**Affiliations:** 1 School of Animal Science and Technology, Shihezi University, Bingtuan Key Laboratory for Efficient Utilization of Non-Grain Feed Resources, Shihezi, Xinjiang, China; 2 Xinjiang Yili Prefecture Animal Husbandry Station, Yining, Xinjiang, China

**Keywords:** high altitude sand area, alfalfa, rhizosphere, core microbiome, networks

## Abstract

Alfalfa cultivation is widely regarded as an effective biological approach for improving desertified and degraded soils. However, it remains unclear how this effect unfolds in high-altitude desert environments and how soil and plant microbiomes assemble when native soil (NS) host plants are replaced. In this study, we tested whether converting NS to alfalfa-cultivated soil (CS) affected the composition of soil microbial communities and changed microbial diversity. Specifically, alfalfa cultivation reshaped core microbial components, increasing beneficial bacteria such as *Arthrobacter* and *Pseudomonas*, potentially enhancing nutrient cycling and plant growth, while reducing certain decomposers (e.g., *Bacteroides*). Results indicated that alfalfa cultivation improved NS quality, and with longer planting time, promoted the recovery and homogenization of soil microbial diversity. This process was accompanied by the replenishment or exclusion of soil microorganisms. Soil organic matter and pH were identified as key drivers of microbial community change. Across 541 bacterial OTUs and 56 fungal OTUs were shared across NS, CS, and alfalfa rhizosphere soil (RS), this core microbiome accounted for a large proportion of reads (bacteria: 28.70%, fungi: 40.37%). Microbial network structure and interactions were more complex NS and RS than in CS, with bacterial interactions more pronounced than fungal ones. Overall, the transition from NS to CS via alfalfa planting positively affected local microbial diversity, and RS assembly was shaped by both recruitment and dispersal. This research highlights the potential of alfalfa to restore high-altitude desertified ecosystems by strengthening microbially mediated soil fertility and biogeochemical cycling, offering insights for sustainable land management in arid regions.

## Graphical Abstract

**Figure:**
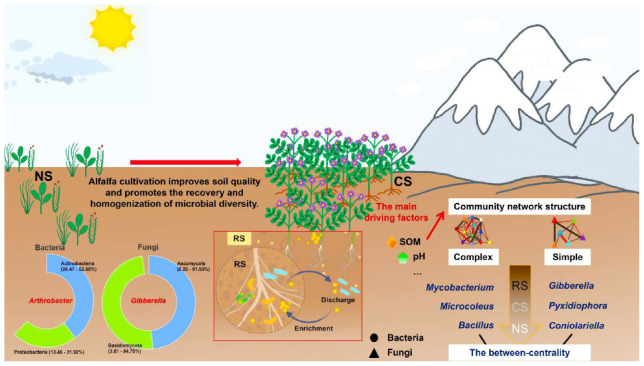
Alfalfa cultivation in high-altitude desertified soils improves soil quality and reshapes bacterial and fungal communities from native soil to cultivated and rhizosphere soils, mainly driven by changes in soil organic matter and pH.

## 1 Introduction

In recent years, under the combined influence of human and natural activities, global climate change has intensified, strengthening the water cycle. These changes, together with water shortages and their profound impacts, are rapidly becoming serious climate and environmental problems worldwide (Huang et al., 2021; Zhao et al., 2014), particularly in fragile, arid, and semi-arid ecosystems (Zhao and Zhang, 2021). At the northern foothills of the Kunlun Mountains in southern Xinjiang, adjacent to the Taklimakan Desert, desertification is particularly severe due to the regionakan Desert, desertification is pa (Xu et al., 2010). The complex and variable regional environment, combined with severe water scarcity, makes it difficult to cultivate many crops effectively and severely threatens the environment that sustains the livelihoods of local farmers and herders (Rumbaur et al., 2015; Zhang et al., 2022). Therefore, protecting and restoring soils in high-altitude sandy areas is a pressing challenge. In high-altitude regions such as the Kunlun Mountains, desertification leads to soil nutrient depletion, reduced microbial diversity, and impaired ecosystem functions, exacerbating biodiversity loss and agricultural challenges (Li and Wei, 2021). Soil microorganisms are pivotal in these ecosystems, driving nutrient cycling, organic matter decomposition, and plant-microbe interactions that support restoration efforts (Wubs et al., 2016; Kang et al., 2021).

*Medicago sativa* L. (alfalfa) is a high-quality forage resource that combines high nutritional value and high yield with soil and water conservation benefits, underscoring its importance for ecological protection and restoration (Ma et al., 2024; Song et al., 2021). The litter and root products of alfalfa plants not only improve soil nutrients but also have significant positive effects on the soil micro-ecological environment (Li et al., 2025; Zeng et al., 2025). As a key component of the soil ecosystem (Wubs et al., 2016), soil microorganisms are closely linked with plants and soil physicochemical properties and mutually constrain one another (Kang et al., 2021; Raaijmakers and Mazzola, 2016). In particular, functions and traits of plant rhizosphere microorganisms (Barret et al., 2011), such as nutrient acquisition, enhanced stress tolerance, protection against soil-borne pathogens, and regulation of host immunity—have far-reaching effects on plants and can even lead to dependence (Berendsen et al., 2012; Mendes et al., 2013). Studies have shown that planting alfalfa in alpine and sandy-soil areas can enrich beneficial microorganisms such as *Arthrobacter* and *Pseudomonas* (Zhao et al., 2022). However, it has also been reported that when the original (uncultivated) soil type is converted to agricultural soil, members of *Bacteroides* with strong abilities to degrade complex polymeric organic matter decline, and overall soil microbial diversity decreases (Ilyas et al., 2022; Wolińska et al., 2017; Chen et al., 2022). For most crops, however, little is known about the interaction and migration between plants and soil microorganisms during cultivation on previously uncultivated land, and whether changes in soil type lead to shifts in microbial diversity and community structure in NS (Chen et al., 2022; Tang et al., 2023).

This study addresses a critical gap: how does alfalfa cultivation in high-altitude desertified soils alter microbial community structure, diversity, and functional potential? Understanding these dynamics is essential for optimizing restoration strategies, as microbial shifts can influence soil fertility, carbon sequestration, and plant resilience in arid environments. Here, we hypothesized that the conversion of native soil (NS) to alfalfa cultivation soil (CS) would improve soil quality, and that alfalfa cultivation would either displace or recruit soil microorganisms, thereby altering microbial community structure and diversity. We further hypothesized that such displacement or recruitment would be more pronounced in rhizosphere soil (RS) and would vary over time. To address these hypotheses, we used 6-month observation intervals to represent a 2-year trajectory. NS, CS and RS samples were collected at high altitudes from severely desertified soil at the northern foot of Kunlun Mountain, followed by microbial community analysis, genus-level abundance modeling, and microbial network analysis.

## 2 Materials and methods

### 2.1 Study area

**FIGURE 1:**
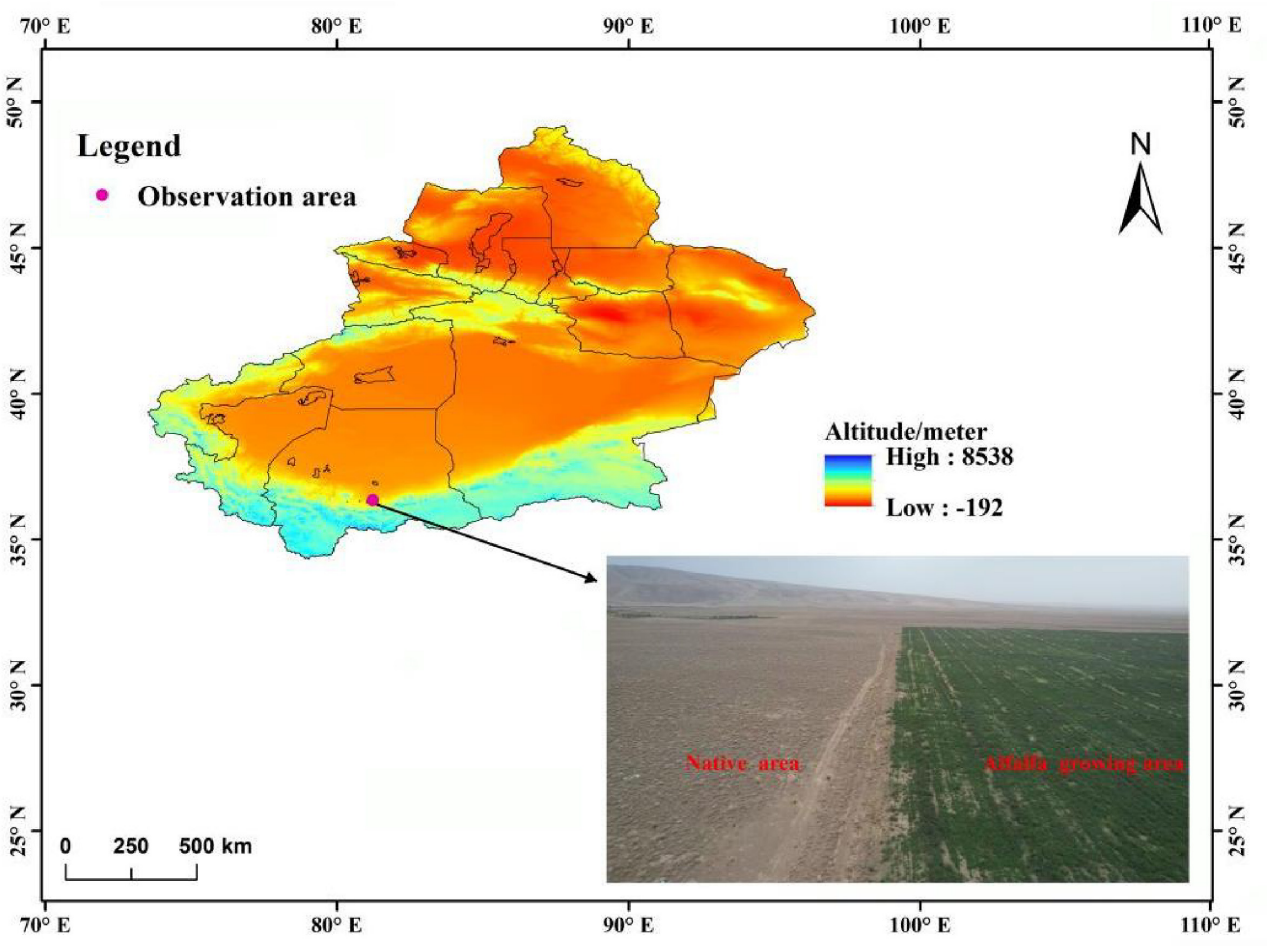
Geographical location of the study area and comparison before and after alfalfa planting.

The research site is located in the desertified area (81°22’ E, 36°25’ N, 2,237–2,305 m) in Bostan Township, Hotan Cele County, Xinjiang, which is situated in the interior of Eurasia and falls within a warm temperate desert and arid climate zone. The native vegetation is temperate alpine desert grassland, and the dominant species include *Reaumuria songonica*, *Ceratoides latens*, *Stipa* spp., and *Seriphidium* spp., with a vegetation cover of 8–10% (Figure 1). The general climate is characterized by high solar radiation and heat, large temperature ranges, low precipitation, high evaporation, an extremely arid atmosphere, and frequent blowing sand (Yu et al., 2018). Temperature and precipitation during the experiment are shown in Supplementary Figure 1.

### 2.2 Experimental design, sample collection, and processing

Alfalfa was planted on September 15, 2020 (autumn-sown). The planting method was mechanical strip sowing with a row spacing of 30 cm. The seeding rate was 22.5–30 kg/hm^2^, and standard dryland practices were used; irrigation was applied only during seedling emergence. A total of 2,000 ha were planted, and the experimental plot covered 6,400 m^2^ (80 × 80 m) and was bordered by 30-cm earthen ridges to reduce human and livestock disturbance. After confirming the successful establishment of alfalfa, we monitored native land and alfalfa fields for two consecutive years (2021–2022) (Wang et al., 2020). No chemical fertilizers or pesticides were applied during the study period to minimize external influences on soil microbial communities. Weeds were controlled manually, twice annually to maintain plot integrity without introducing herbicides.

We conducted four 6-month-interval samplings across two growing seasons to resolve rhizosphere-to-periphery spatial variation with consistent seasonal timing. Supplementary sowing after year-2 harvest for higher forage yield prevented further long-term sampling. native soil (NS, vegetation in areas where alfalfa is not planted), alfalfa cultivated soil (CS, between the rows of alfalfa plants), and alfalfa rhizosphere soil (RS, soil attached to alfalfa roots is collected by the root shaking method) (Qi et al., 2023) were collected at: April 2021 (21-I), October 2021 (21-II), April 2022 (22-I), and October 2022 (22-II). A sampling depth of 0–40 cm was maintained. After screening and selection, the collected soil samples were divided into two parts: One was stored in air-dried bags for determination of soil physical and chemical properties, and the other was placed into a foam box with dry ice and stored in an ultra-low temperature refrigerator (–80°C) for analysis of soil microbial communities.

### 2.3 Soil physicochemical characteristic analyses

Bulk density (BD) was measured using the core method (100 density (BD) was measured usin. Soil water content (SWC) was measured by oven-drying fresh samples at 105°C for 48 h. Soil pH was determined with a digital pH meter at a 1:2.5 soil:water ratio, and electrical conductivity (EC) was measured at a 5:1 water:soil ratio. Soil organic matter (SOM) was analyzed by the potassium dichromate volumetric method. Total nitrogen (TN) was quantified using sulfuric acid digestion and the micro-Kjeldahl method, while alkali-hydrolytic nitrogen (AN) was assessed by alkali-hydrolysis diffusion (Kjeldahl, 1883). Total phosphorus (TP) was determined via the molybdenum-antimony anti-colorimetric method (HCLO_4_–H_2_SO_4_), and available phosphorus (AP) was extracted with sodium bicarbonate and measured by spectrophotometry. Total potassium (TK) was determined by NH*4*OAc extraction-flame photometry, and available potassium (AK) was measured using ammonium acetate leaching-flame photometry (Bao, 2005). Urease activity (S_EU) was measured following Gu et al. (2009) after 24TERO_ITEM CSL_CIT37°C. Phosphatase (S_NP) and sucrase (S_SC) activities were determined by colorimetry as described by Guan (1986). Catalase activity (S_CAT) was assayed as in Trasar-Cepeda et al. (1999). All samples were analyzed in quintuplicate.

### 2.4 Soil DNA extraction and gene sequencing

Soil DNA was extracted using a soil DNA extraction kit (Omega Bio-Tek, Norcross, GA, United States), followed by electrophoresis on 1% agarose gels to assess the quality of the total DNA extraction and determine the total DNA concentration. PCR was performed on an ABI GeneAmp^®^ 9700 thermal cycler (Applied Biosystems) to amplify the V3 (Applied Biosystems) med on an ABI GeneAmp6S rRNA gene and the fungal ITS1 region from soil samples. The primers used were 338F: (5’-ACTCCTACGGGAGGCAGCAG-3’), 806R: (5’-GGACTACHVGGGTWTCTAAT-3’), ITS1F: (5’-CTTGGTCATTTAGAGGAAGTAA-3’), and ITS2R: (5’-GCTGCGTTCTTCATCGATGC-3’). PCR amplification conditions included pre-denaturation at 95°C for 3 min; 35 cycles of denaturation at 95°C for 30 s, annealing at 55°C for 30 s, and extension at 72°C for 30 s. PCR products (AxyPrep DNA recovery kit from AXYGEN) were recovered and detected by 2% agarose gel electrophoresis. The PCR products were sent to Shanghai Magi Biomedical Technology Co., Ltd. for sequencing on the Illumina MiSeq PE300 platform. We processed reads with QIIME (1.9.1) and UCHIME, then annotated bacteria with SILVA (release 138) and fungi with UNITE (release 8.3), these current releases provide larger, better-curated references and updated taxonomy, thereby improving reliability and depth of taxonomic calls (Nilsson et al., 2019; Quast et al., 2013). UPARSE (7.0.1090) was used to cluster the valid sequences of all samples at a 97% similarity level, and the Silva and Unite databases were used to annotate the representative sequences of OTUs, respectively, with a comparison threshold of 70% (Nilsson et al., 2019).

### 2.5 Statistical analyses

Statistical analyses were performed using SPSS (version 22.0; SPSS Inc., Chicago, IL, United States), with Duncan’s multiple comparison test, and *P* < 0.05 was considered significantly different. The minimum data set (MDS) method was adopted to calculate the soil quality index: Principal component analysis was conducted on the soil indicators. Then, MDS of soil quality indicators was constructed based on the Norm value and Pearson correlation analysis. Finally, the membership function was used to calculate the membership degree of the soil indicators. Finally, the soil quality index (SQI) is calculated based on the membership degree and weight of the obtained soil indicators (Raiesi, 2017).


$$N_{i\,k}=\sqrt{\overset{k}{\underset{i=1}{\sum }}\left(u_{i\,k}^{2}\cdot \lambda_{k}\right)}$$

In the formula, N_ik_ represents the combined load of the i-th variable on the first k principal components with eigenvalues > 1, that is, the Norm value of the i-th variable. u_ik_ is the rotation factor load of i variables on the k-th principal component, and λ_k_ is the eigenvalue of the k-th principal component.


$$\text{SQI}=\overset{n}{\underset{i=1}{\sum }}w_{i}\,S_{i}$$

In the formula, S_i_ represents the membership degree of the i-th soil indicator, W_i_ represents the weight value of the indicator, and n represents all evaluated indicators/the minimum dataset indicator. The higher the SQI value, the better the soil quality.

The sequencing data of the bacterial and fungal communities were analyzed using the online Majorbio Cloud Platform.^1^ Shannon, Sobs (observed richness), and Chao1 diversity indices were calculated using the boot (v1.3.18) and stats (v3.3.1) packages in R (v3.3.1), and one-way ANOVA with TukeyKramer *post-hoc* test was applied, *P*-values were adjusted using false discovery rate (FDR) correction. β-diversity was assessed using principal coordinate analysis (PCoA), of Bray–Curtis dissimilarities and tested with ANOSIM (vegan, R). Then, using the stats package in R (v3.3.1) and the SciPy package in Python (v1.0.0), the Kruskal–Wallis rank sum test, differential abundance analysis, FDR multiple-testing correction, and uncorrected Welch’s *post-hoc* test (uncorrected) were performed for the 12 treatment combinations (three soil types × sampling times). R (v3.3.1) generated the Venn diagrams representing the exclusive and shared genera and to produced community bar plots based on the data table in the tax_summary_a folder. The selected OTU table was used for core microbiome analysis and R (v3.3.1) was used for plotting. NetworkX (v1.11) was used to analyze the correlation network, selecting the statistically significant correlations (*P* < 0.01) with | r| ≥ 0.85 and *R* (v3.3.1) was then used to visualize the network.

## 3 Results

### 3.1 Planting alfalfa improves the quality of NS

Analyzing NS, alfalfa-cultivated soil (CS), and alfalfa rhizosphere soil (RS), we found that these soils differed in physicochemical properties. In brief, compared with NS, CS had higher SOM, AP, AN, and AK, and higher pH and EC. This pattern was more pronounced in RS (Supplementary Table 1). The minimum data set (MDS) reduces data redundancy and can effectively replace the complete dataset to evaluate topsoil quality in the study area. Principal component analysis (PCA) was conducted on 16 soil indicators, and five principal components with eigenvalues > 1 were selected, with a cumulative contribution rate of 79.307%. Ultimately, six indicators—SOM, TP, BD, S_NP, EC, and AP—were selected as the MDS. Soil quality analysis indicated that alfalfa cultivation significantly improved soil quality (Table 1).

**TABLE 1:** Soil quality index for three types of soil and four periods.

Item	Native soil	Cultivate soil	Rhizosphere soil
21-I	0.38 ± 0.11Ba	0.63 ± 0.17Aa	0.67 ± 0.14Aa
21-II	0.37 ± 0.14Ba	0.69 ± 0.18ABa	0.59 ± 0.31Aa
22-I	0.26 ± 0.14Bab	0.47 ± 0.18ABa	0.61 ± 0.09Aa
22-II	0.16 ± 0.07Bb	0.45 ± 0.11Aa	0.48 ± 0.09Aa

Values are means ± SD. Analyses were performed separately for each soil at each sampling time using ANOVA with Duncan’s test (*P* < 0.05). Uppercase letters denote significant differences among soils within the same sampling time; lowercase letters denote differences among sampling times within a soil. 21-I, first sampling (Apr. 2021); 21-II, second sampling (Oct. 2021); 22-I, third sampling (Apr. 2022); and 22-II, fourth sampling (Oct. 2022).

### 3.2 Alfalfa planting changed the diversity of soil microbial community

**FIGURE 2:**
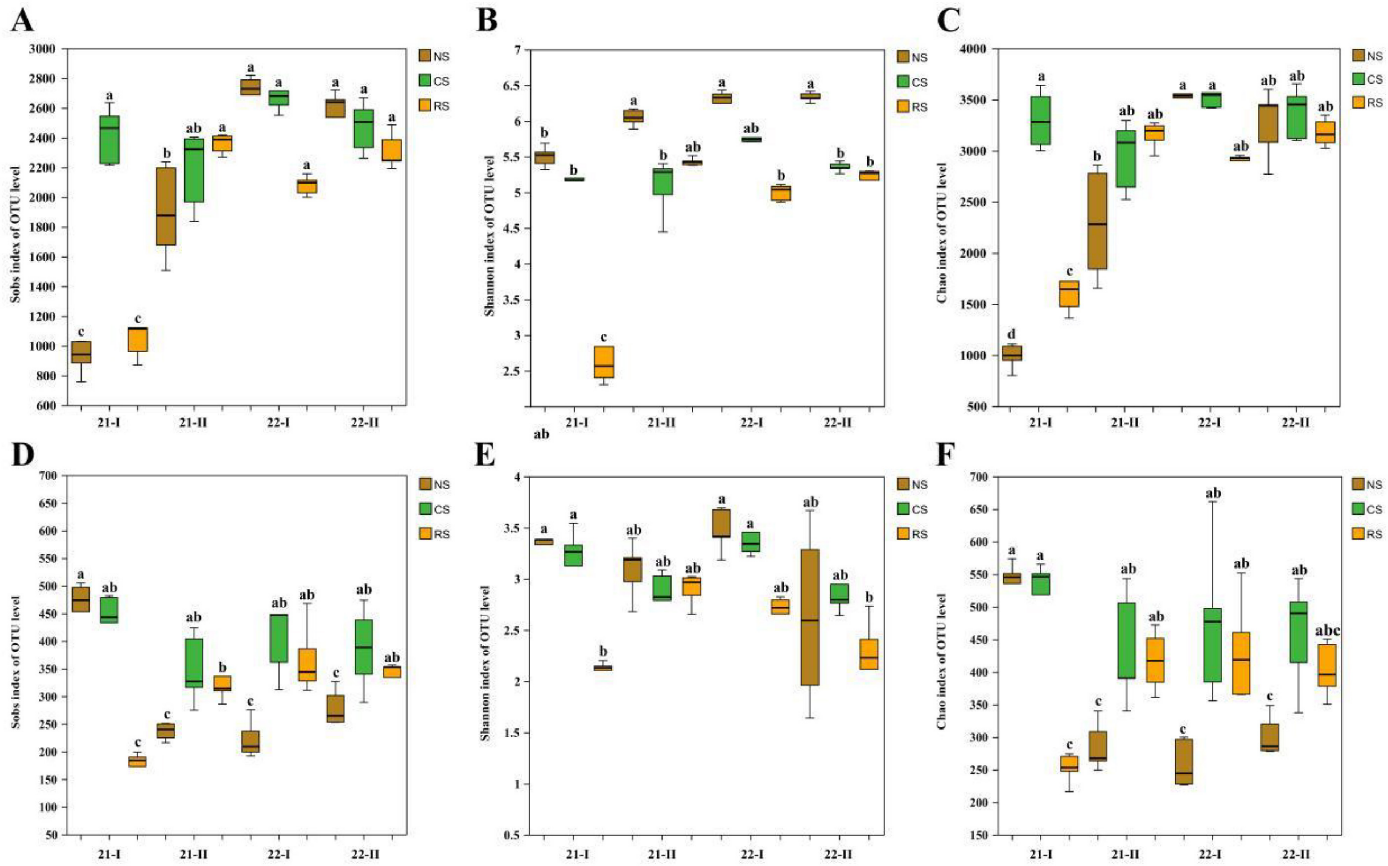
Comparative analysis of 16S rRNA gene and ITS sequence α diversity of bacteria and fungi in three soils. **(A–C)** Sobs, Shannon, Chao1 of bacterial communities. **(D–F)** Sobs, Shannon, Chao1 of fungal communities. Effects of soil type and time were tested by one-way ANOVA (*P* < 0.05). Different lowercase letters indicate significant differences among treatments. NS, native soil (brown); CS, cultivated soil (green); and RS, rhizosphere soil (orange). 21-I (Apr. 2021), 21-II (Oct. 2021), 22-I (Apr. 2022), 22-II (Oct. 2022).

After mass filtration, 4,116,016/9,220,384 optimized bacterial/fungal sequences were recovered. The average sequence length was 413/233 bp, which represents 12,037/2,795 OTUs with 97% sequence similarity. The sequencing depth reflected by the sparse curve indicated the reliability of the data (Supplementary Figure 2). For α diversity, bacteria (Figures 2A–C), the Sobs index showed clear spatial differences at the first sampling (21-I), with RS lower than NS, followed by increases in RS that narrowed between-soil differences across the four campaigns. The Shannon index likewise indicated that RS had the lowest diversity at 21-I but increased over time. The Chao1 index revealed a temporal rise in richness in NS and RS, tending toward convergence with CS by 22-II. For fungi (Figures 2D–F), the Sobs index showed an initial separation at 21-I, after which RS largely tracked CS and differences diminished over time. The Shannon index was lowest in RS at 21-I but increased during subsequent samplings. The Chao1 index indicated that richness dynamics were opposite in RS and CS, with temporal changes reducing differences between-soils by 22-II.

**FIGURE 3:**
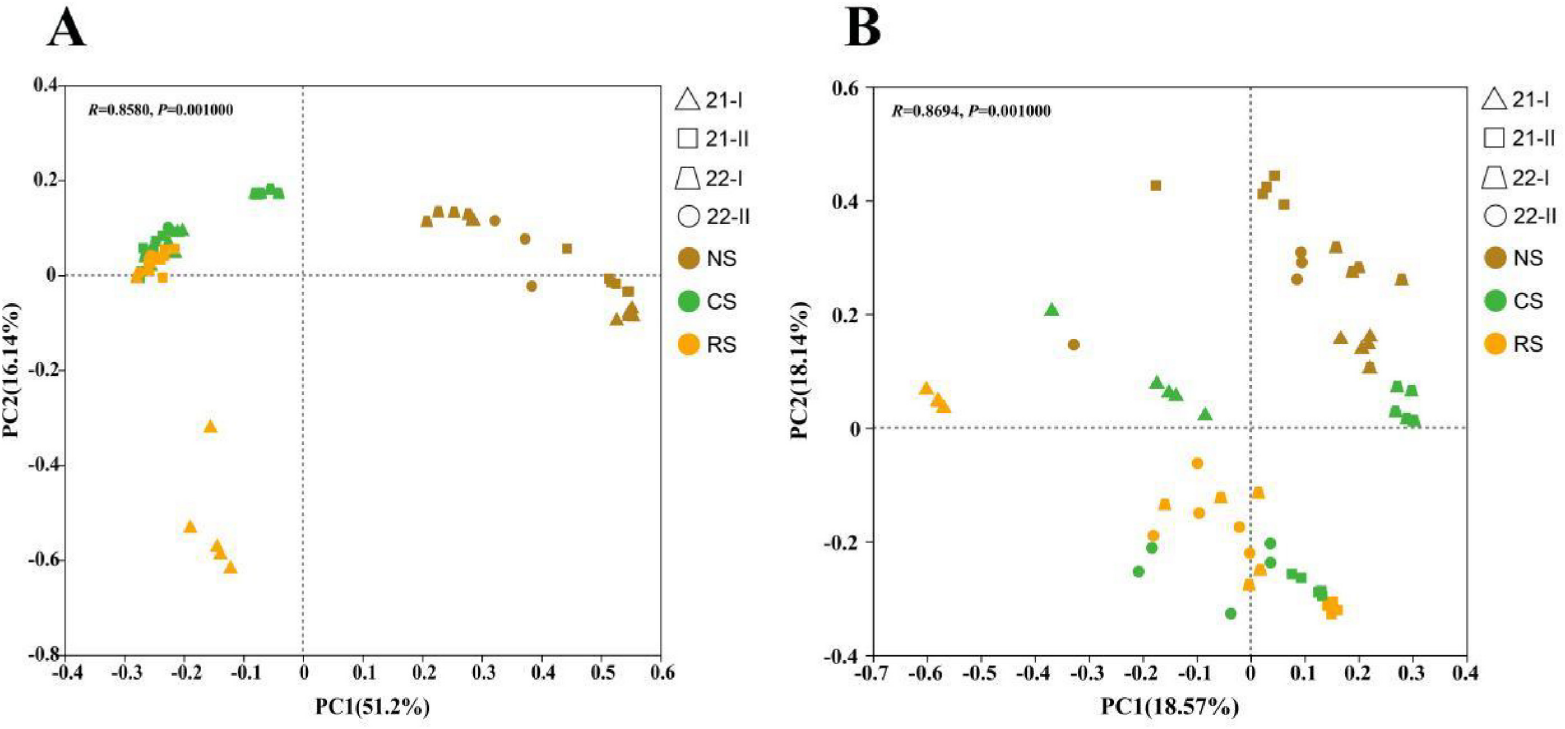
PCoA of community structure across three soils and four sampling times. **(A)** 16S rRNA gene (bacteria). **(B)** ITS (fungi). NS, native soil (brown); CS, cultivated soil (green); and RS, rhizosphere soil (orange). Triangle 21-I: (Apr. 2021), rectangle 21-II: (Oct. 2021), trapezoid 22-I: (Apr. 2022), circular 22-II: (Oct. 2022). Bray–Curtis distances were computed from CSS-normalized counts.

For β-diversity, PCoA of Bray–Curtis dissimilarities showed a significant effect of soil type (Figure 3A). Soil type alone explained 67.34% of the total variation in bacterial community composition and 37.71% of fungal community composition. (ANOSIM, *P* < 0.01). Subsequently, sampling times for the same soil type separately (Supplementary Figure 3). For bacteria, time explained 62.96% in NS, 55.42% in CS, and 80.41% in RS (ANOSIM, *P* < 0.01). For fungi, time accounted for 48.91% in NS, 60.62% in CS, and 72.64% in RS. (ANOSIM, *P* < 0.01) (Figure 3B).

### 3.3 Specific differences of microorganisms in different soil types

**FIGURE 4:**
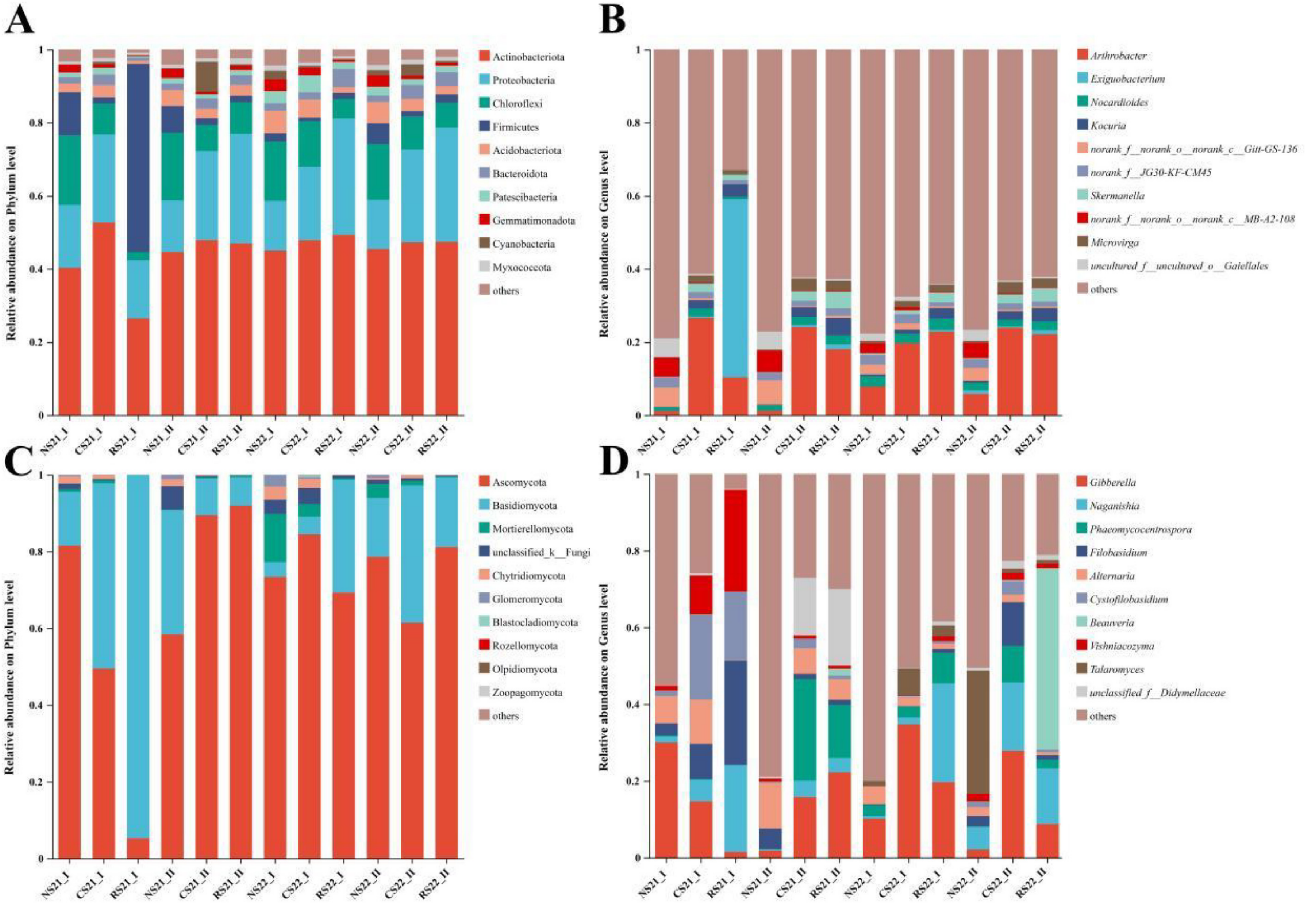
Relative abundance of dominant bacterial and fungal taxa across three soil types. Stacked bar charts display taxa whose total relative abundance exceeds 1% in at least one sample. **(A)** Bacterial phyla; **(B)** bacterial genera; **(C)** fungal phyla; **(D)** fungal genera. NS, native soil; CS, alfalfa-cultivated soil; RS, alfalfa rhizosphere soil. 21-I (Apr. 2021), 21-II (Oct. 2021), 22-I (Apr. 2022), 22-II (Oct. 2022).

The changes of α- and β-diversity of bacterial and fungal communities detected in soil showed by alfalfa planting in time prompted us to explore the differences of taxonomic identity and relative abundance of microorganisms in each soil (Figure 4). Among bacteria, Actinobacteriota (26.47–52.65%), Proteobacteria (13.45–31.92%), and Chloroflexi (2.20–19.06%) were the most abundant phyla, while the fungi in this area were mainly Ascomycota (5.20–91.93%) and Basidiomycota (3.81–94.76%). Next, we compared the differences in bacteria and fungi across sampling times for the three soil types (Supplementary Figures 4, 5). For bacteria, RS showed higher Firmicutes at 21-I, and higher Proteobacteria at 21-II, whereas Chloroflexi and Acidobacteriota displayed transitional trends. At the genus level, *Exiguobacterium* was significantly enriched in RS at 21-I; *Nocardioides* increased from NS to CS but was absent in RS at 21-I; and *Microvirga* appeared in CS and RS. In subsequent samplings, *Nocardioides*, *Skermanella*, and *Microvirga* were consistently higher in CS and RS than in NS. For fungi, NS initially had higher Ascomycota while RS had higher Basidiomycota at 21-I. At 21-II, CS and RS were enriched in *Phaeomycocentrospora* and *Naganishia*; at 22-I and 22-II, RS remained enriched in *Naganishia*, whereas CS was enriched in *Gibberella*. *Talaromyces* increased in CS and RS at 22-I but decreased at 22-II, and *Beauveria* was enriched in RS (Kruskal–Wallis test, *P* < 0.05, FDR-corrected).

### 3.4 Study on microbial diversity of three soils at different sampling times

**FIGURE 5:**
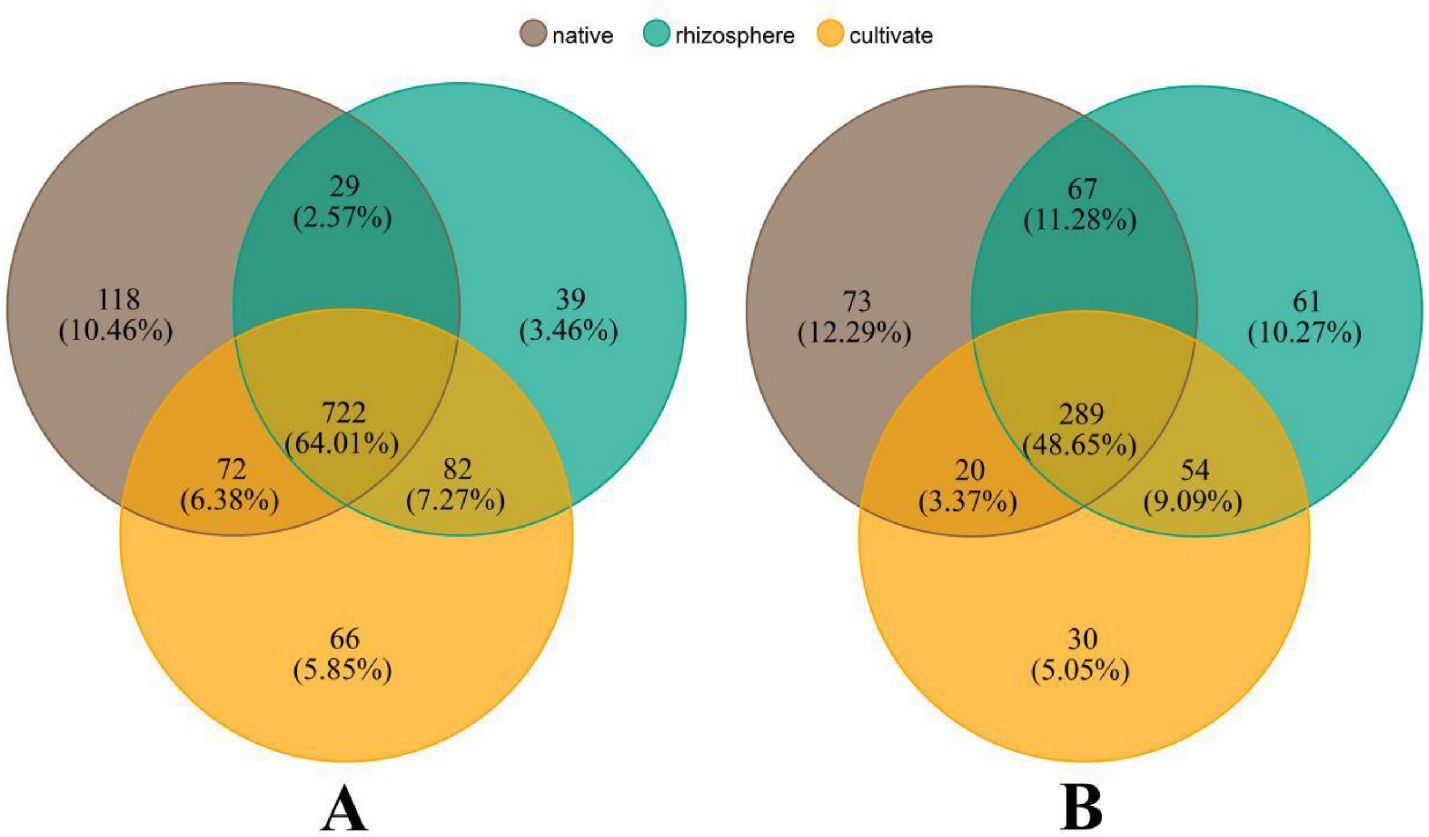
The exclusive and shared genera in three soils. native, Native soil (brown); cultivate, cultivated soil (green); and rhizosphere, rhizosphere soil (orange). **(A)** Bacterial community. **(B)** Fungal community.

To clarify the spatial and temporal changes in soil microbial diversity after alfalfa planting, we used Venn diagrams to summarize “endemic” (defined here as genera detected only in one soil type across the 2-year sampling) and shared genera across NS, CS, and RS (Figure 5). The results showed that there were 64.01%/48.65% bacterial/fungal genera in the three soils, with the “endemic genera” were most numerous in NS and fewest in RS. Over time, the set of shared genera between CS and RS exceeded that shared with NS, and the composition of “endemic genera” shifted accordingly (Supplementary Figure 6). The shared genera of bacteria and fungi in CS and RS were more abundant than those in NS, but the “endemic genera” were different. In summary, the genera of both bacteria and fungi were more abundant in NS than in CS and RS. Additionally, it should be noted that the abundance of these “endemic genera” in the three soils was relatively low. Compared to fungi, bacteria exhibited a more pronounced trend.

### 3.5 Analysis of core microorganisms in three soils

Of the 12,037 bacterial and 2,795 fungal clustered OTUs, 541 and 56 OTUs, respectively, were present in all three soils at all sampling times. These OTUs, classified at the genus level, comprised 4.49% of all bacterial OTUs and 2.00% of all fungal OTUs, yet represented 28.70 and 40.37% of total bacterial and fungal sequences, respectively. The bacterial core microbiome, comprised OTUs assigned to Actinobacteria (*n* = 199), Proteobacteria (*n* = 127), Chloroflexi (*n* = 106), and Firmicutes (*n* = 13), together accounting for 85.18% of the mean relative abundance; with *Arthrobacter* as the most abundant contributor (two OTUs, 16.99%). The core microbiome of fungi comprised 38 Ascomycota (68.57%) and 12 Basidiomycota (26.35%) OTUs, with *Gibberella* as the most abundant contributor (two OTUs, 18.63%). We also analyzed soil-specific cores to assess how alfalfa-driven changes in soil type contributed to the overall communities.

**FIGURE 6:**
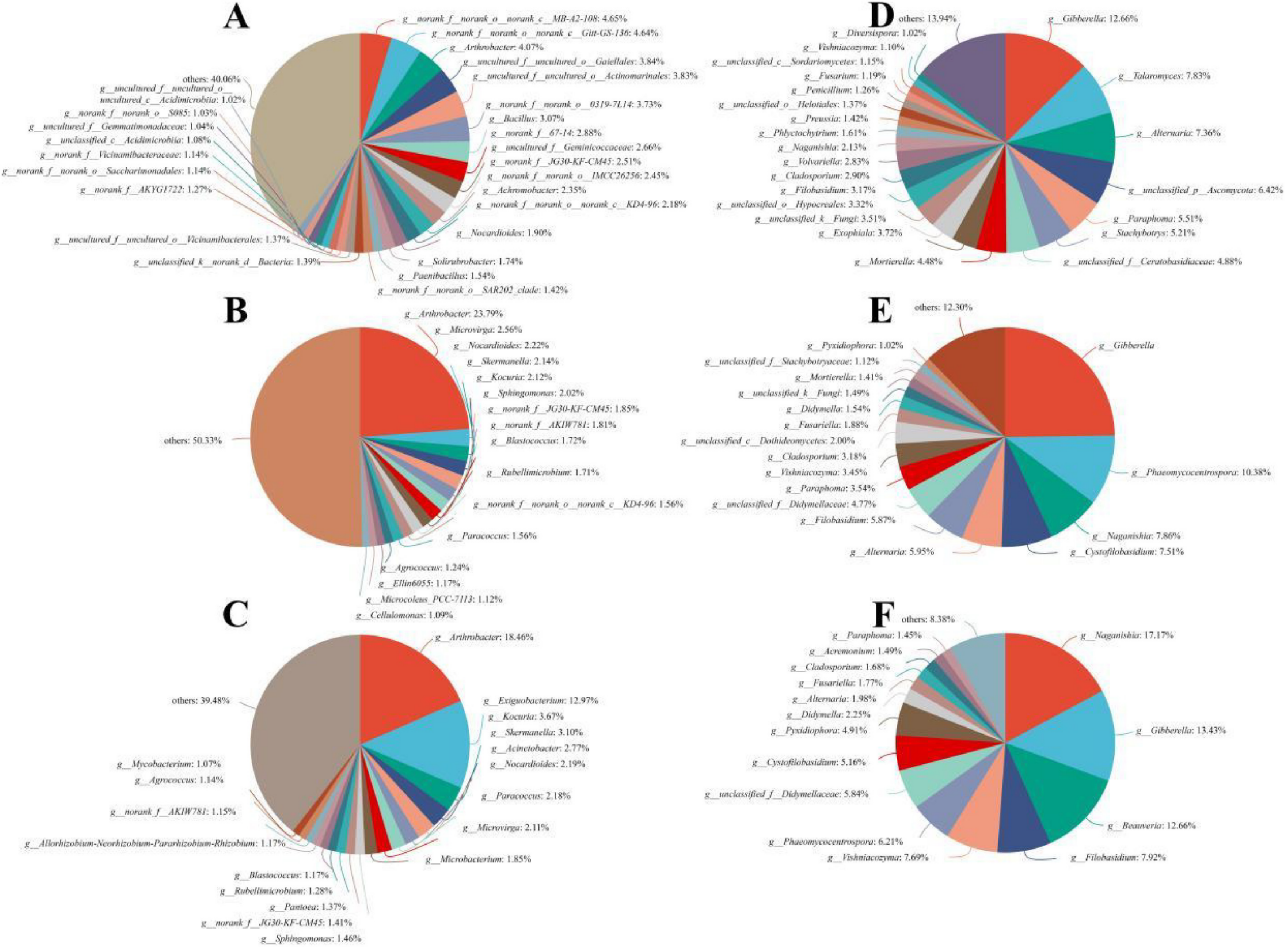
Core genera in bacterial and fungal communities across three soil types. **(A–C)** Bacterial communities in native soil (NS), cultivated soil (CS), and rhizosphere soil (RS). **(D–F)** Fungal communities in NS, CS, and RS. All taxa are shown at the genus level, and percentages indicate relative abundance within each soil type.

The core bacteria in NS, CS, and RS included 1,475, 1,475, and 1,720 OTUs, respectively (Figure 6), accounting for 12.25, 19.63, and 14.29% of total OTUs and 34.75, 37.85, and 35.37% of total reads, respectively. Core fungi included 145 OTUs in NS, 242 OTUs in CS, and 164 OTUs in RS, accounting for 5.19, 8.66, and 5.87% of total OTUs and 57.22, 47.09, and 46.43% of total reads, respectively. Acidobacteria, Chloroflexi, and Proteobacteria were the dominant phyla among the three soil core bacteria, while *Ascomycota* and *Basidiomycota* were the dominant phyla of the core fungi in NS and RS. In CS, taxa annotated as unclassified_k__Fungi were also part of the core fungal community. Phylum-level proportions of these core groups differed among soils. The dominant bacteria changed from *g_norank* in NS to *Arthrobacter* in CS and RS at the genus level, while the dominant fungi changed from *Gibberella* in NS and CS to *Naganishia* in RS.

### 3.6 Correlation network analysis

**FIGURE 7:**
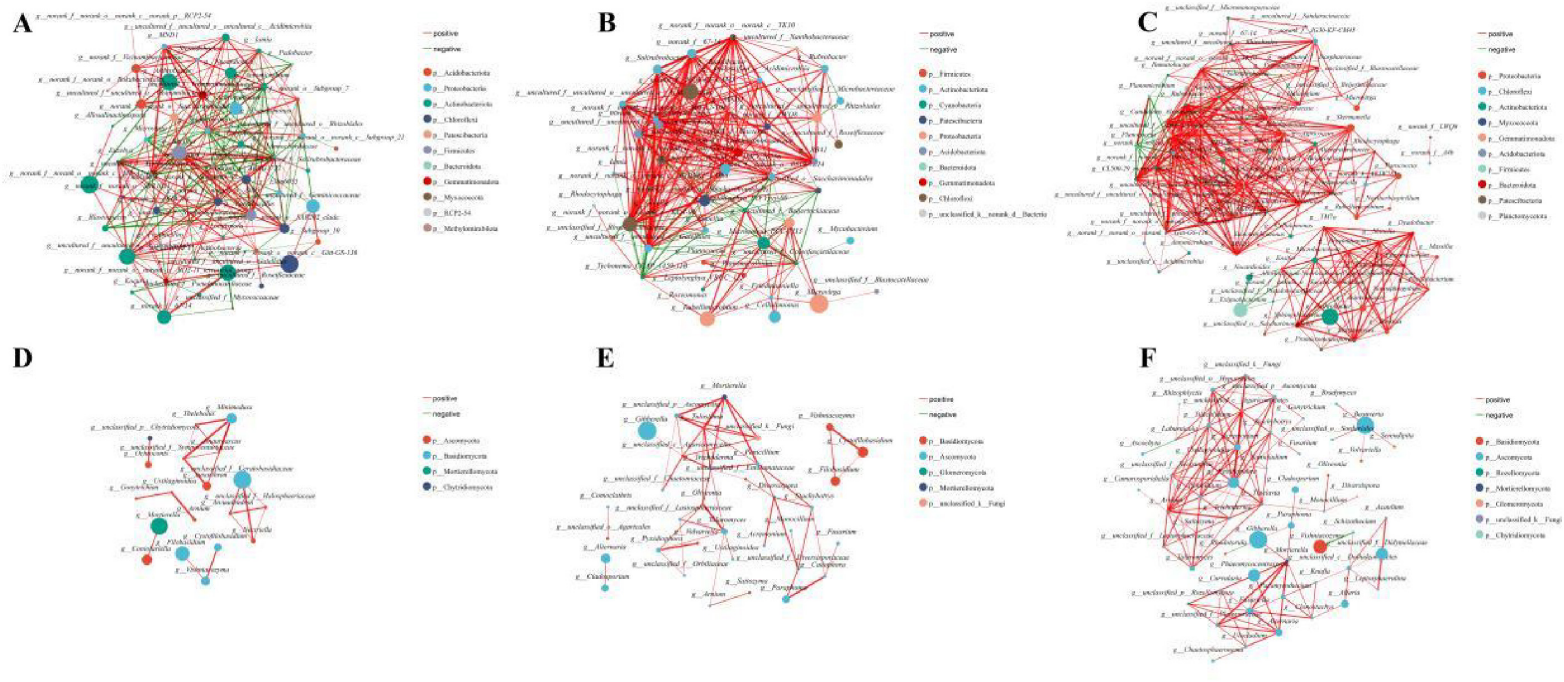
Correlation network across three soils. **(A–C)** Bacteria: NS (native soil), CS (cultivated soil), RS, (rhizosphere soil). **(D–F)** Fungi: NS, CS, RS. Edges (Spearman): red: positive, green: negative; thicker lines indicate stronger correlations.

Correlation (Spearman) network analysis was used to assess to evaluate the complexity of the interactions between bacterial and fungal communities in soils under alfalfa cultivation. Pairwise Spearman correlations among taxa occurrences were computed (Figure 7). Networks were inferred and visualized, and standard topological metrics were calculated (Supplementary Table 2). Across soils, bacterial co-occurrence networks had more nodes and significant edges, with more densely interconnected modules. Fungal networks comprised more modules but were sparser, with fewer OTUs per module. As summarized in Supplementary Table 2 the number of nodes, edges, network density, and average number of neighbors in CS were lower, but the characteristic path length was higher, for both bacteria and fungi. We used betweenness centrality (BC) to identify key genera; those with higher BC included: NS, bacteria: *Bacillus* (OTUs 5,264, 5,122, 5,073, 1,976); fungi: *Coniolariella* (OTUs 812, 869, 2,034). CS, bacteria: *Microcoleus* (OTU 1,952), *Rubellimicrobium* (OTUs 2,396, 2,358); fungi: *Pyxidiophora* (OTU 728). RS, bacteria: *Mycobacterium* (OTUs 1897, 335); fungi: *Gibberella* (OTUs 921, 971).

### 3.7 Correlation analysis of environmental factors

**FIGURE 8:**
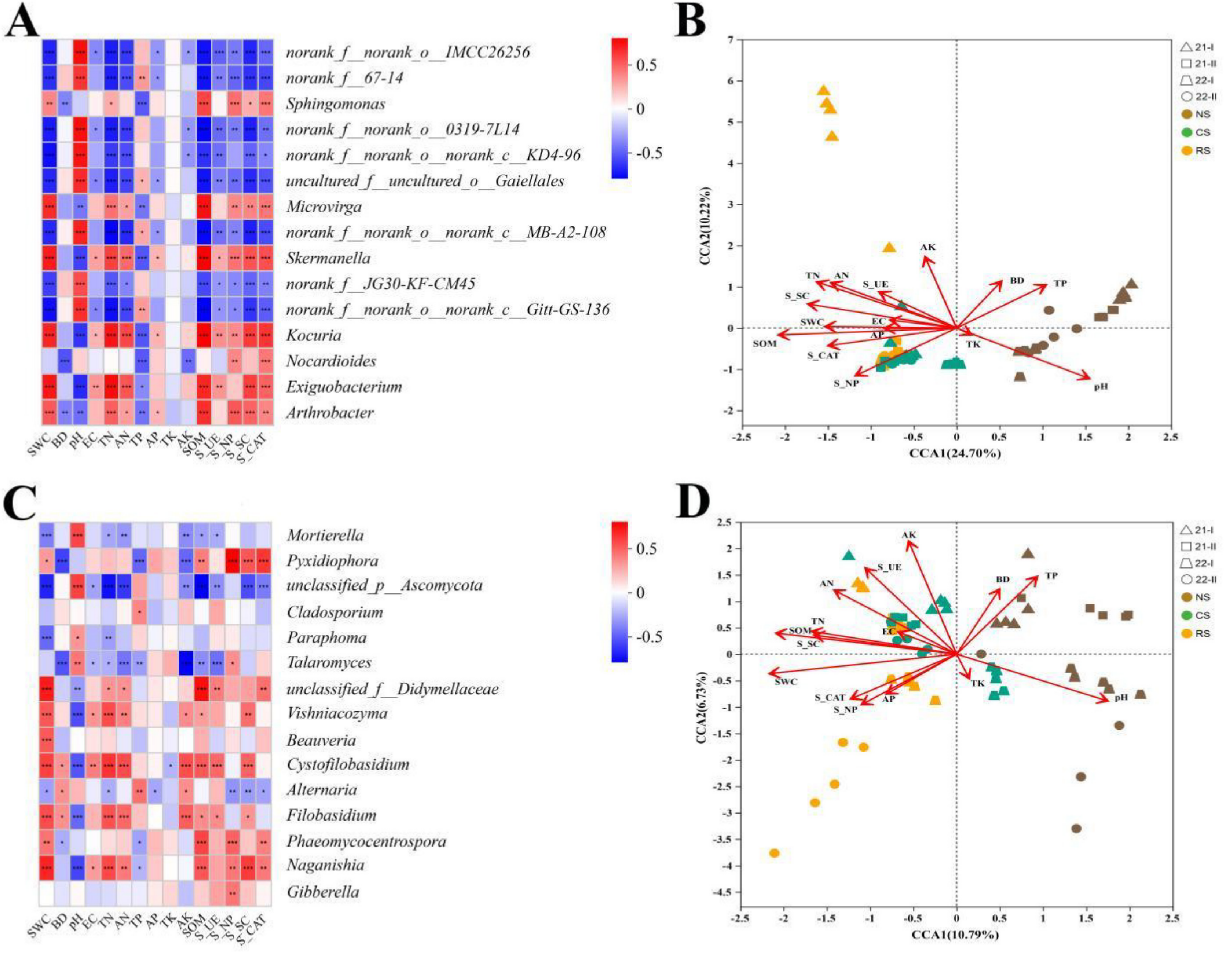
Genus-level relative-abundance correlations with environmental variables and CCA ordinations. **(A,C)** Spearman correlations—bacteria; fungi. **(B,D)** CCA—bacteria; fungi. Soils: NS (brown), CS (green), RS (orange). Sampling times: 21-I (Apr. 2021), 21-II (Oct. 2021), 22-I (Apr. 2022), 22-II (Oct. 2022).

Spearman rank correlations indicated that, among the dominant bacteria, classified genera such as *Arthrobacter*, *Exiguobacterium*, *Nocardioides*, and *Kocuria* were significantly positively associated with most soil variables (*P* < 0.05), while unclassified genera showed negative associations (*P* < 0.05). However, soil pH showed the opposite trend (Figure 8A). For fungi, most of them showed a significant positive correlation with soil enzyme activity and SWC (*P* < 0.05), whereas *Alternaria*, *Talaromyces*, *Paraphoma*, and *Mortierella* showed the opposite trend. Notably, there was a significant negative correlation between soil pH and fungi belonging to *Basidiomycetes* (Basidiomycota) (*P* < 0.05) (Figure 8C). Non-dominant bacterial taxa showed heterogeneous relationships with environmental variables. CCA revealed that axes 1 and 2 explained 24.70 and 10.22% of the variation in bacteria (cumulative 34.92%) and 11.05 and 6.81% in fungi (cumulative 17.86%), indicating that microbial communities were shaped by physicochemical gradients. For the first axis, the most important environmental factors for bacteria were SOM (*r*^2^ = 0.6741, *P* < 0.01), sucrase activity (S_SC) (*r*^2^ = 0.4609, *P* < 0.01), TN (*r*^2^ = 0.4545, *P* < 0.01), and pH (*r*^2^ = 0.4316, *P* < 0.01) (Figure 8B). For fungi, SWC (*r*^2^ = 0.6412, *P* < 0.01), SOM (*r*^2^ = 0.6007, *P* < 0.01), AK (*r*^2^ = 0.5122, *P* < 0.01), and pH (*r*^2^ = 0.49, *P* < 0.01) had significant influences on the distribution of soil microbial communities (Figure 8D).

## 4 Discussion

### 4.1 Analysis of microbial community diversity and driving factors

Our results show that alfalfa planting initially reduced microbial diversity in the rhizosphere but led to its gradual recovery and homogenization over time, consistent with the positive impacts on soil quality. This initial decline may reflect selective pressure from alfalfa root exudates, favoring beneficial microbes while disfavoring others, ultimately enhancing ecosystem stability (Berendsen et al., 2012; Pérez-Jaramillo et al., 2019). Compared to NS, alfalfa planting slowed the decline in soil microbial richness and modestly improved soil fertility. Genus-level analyses showed that differences in dominant genera among the three soils were most pronounced at the first sampling, likely reflecting shifts following replacement of native vegetation in NS. For example, *Exiguobacterium*, which promotes nitrate transformation and contributes to bioremediation (Kasana and Pandey, 2018), showed increased abundance in the RS, promoting early colonization by alfalfa. As a dominant bacterium in alpine soils, *Arthrobacter* is frequently reported to be involved in nitrogen fixation (Jiang et al., 2022). Furthermore, the dominant genera of fungi, such as *Gibberella* and *Naganishia*, shifted over time, indicating that microbial activity is constrained by certain factors (Zhao et al., 2014; Zhao and Zhang, 2021).

Soil factors, as mentioned in the CCA, were the primary selection pressures in alfalfa soils (Yu et al., 2024). For example, alfalfa consumes large amounts of nitrogen and phosphorus during its growth. Although these nutrients were supplemented early, their contents in alfalfa soils remained only slightly above NS. These nutrients have a significant influence on the composition of the soil microbial community (Van Der Heijden et al., 2016; White et al., 2021), and the soil microenvironment is regulated by plant genetic background and metabolism, which in turn shapes microbial community structure. According to the correlation analysis, SOM and pH significantly influenced community distribution. Notably, pH is a key driver; diversity is typically lower in acidic soils (Kuramae et al., 2012; Fierer and Jackson, 2006). Although alfalfa was managed under dryland conditions, the substrate added to NS in 2020 to ensure establishment may confound this inference, potentially driving genus-level differences (Zhu et al., 2022; Geisseler and Scow, 2014). The results further showed that compared with NS, both CS and RS drove larger, time-dependent shifts in microbial community assembly because continuous inputs of root exudates and decomposing plant residues increased labile C and nutrients and altered microsite conditions (e.g., pH, SOM, moisture), thereby strengthening plant-mediated environmental filtering. One potential mechanism is this native vegetation in NS was replaced by alfalfa, which takes time to adapt (Wang et al., 2022). For alfalfa, the harsh abiotic conditions in the NS may transiently alter the quantity and quality of root exudates (Chen et al., 2022; Doornbos et al., 2012). However, physical properties of NS changed little and were likely influenced mainly by temperature given the low vegetation cover (Zhao and Zhang, 2021). Beyond structural changes, alfalfa cultivation likely influences microbial functions. For instance, enriched *Arthrobacter* and *Pseudomonas* in CS and RS are known for phosphate solubilization and nitrogen fixation, potentially improving nutrient availability and plant growth (Zhao et al., 2022). Reduced *Bacteroides* may decrease complex organic matter decomposition rates initially, but increased network complexity in RS suggests enhanced functional redundancy and resilience in biogeochemical cycles (Wolińska et al., 2017; Mendes et al., 2013). Additionally, soil in this study resemble other high-altitude desertified soils (Huang et al., 2021; Ilyas et al., 2022), with low available N, P, and organic matter. These conditions are unfavorable for establishing and functioning legume-microbe symbioses, constraining N acquisition, reducing root exudation, and ultimately limiting legume growth.

### 4.2 Analysis of microbial community structure and core genera

In this study, NS harbored more unique bacterial and fungal genera than CS and RS, with a clear turnover from NS to CS/RS. The genera endemic to NS included *Microbulbifer* and *Septoglomus*. *Microbulbifer* is commonly found in marine environments and plays a key role in carbohydrate metabolism with bioactive potential (Huang et al., 2020). *Septoglomus* is a dominant roots symbiont of alfalfa, pepper, onion, and maize in arid regions (Guardiola-Marquez et al., 2022). Inoculation with spores of *Septoglomus deserticola* alleviates drought stress in soybean, increasing seed soluble sugar, lipid, protein, and oil (Sheteiwy et al., 2021). Thus, *Septoglomus* may facilitate alfalfa growth under the conditions of this study. *Oscillatoria* and *Ascotricha* were found exclusively in CS, and are often associated with heavy-metal–influenced environments. These metals were likely introduced during fertilizer production and application to CS, potentially explaining their occurrence as CS endemics. Both *Oscillatoria* and *Ascotricha* produce antibacterial secondary metabolites (Bishoyi et al., 2024; Chiranjeevi et al., 2022), which may help protect plants from biotic stress caused by soil-borne pathogens. However, further experiments are needed to clarify the effects of these enriched microorganisms on alfalfa growth and health. The endemic microbes in RS included *Mariniluteicoccus* and *Phaeosphaeriopsis*. Members of these groups and other rhizosphere taxa can degrade lignocellulose and produce antimicrobial metabolites (Barka et al., 2016; Phookamsak et al., 2014; Zhong et al., 2022), thereby benefiting C and N transformations associated with alfalfa. Notably, some unclassified bacteria were restricted to specific soils and comprised a substantial fraction. The origins and drivers warrant further study. Additionally, whether beneficial microbes with antibacterial activity, such as *Septoglomus* and *Cordyceps* (Yue et al., 2013), found only in NS, can be reintroduced into CS and RS to enhance alfalfa growth is a key direction for future research.

In general, the core microbial taxa shared across the three soils were abundant. At the genus level, most of these microbes promote plant growth, solubilize phosphorus, transform nitrogen, and tolerate extreme conditions as reported similar habitats (Zhao et al., 2022; Yao et al., 2019). We further observed that, relative to NS, rhizobia became more evident in CS and RS—an expected feature of legume systems. These rhizobia were also present in NS, but their relative abundance was low. Alfalfa rhizodeposits may enhance their responsiveness to root signals, promoting faster adaptation and growth (Berendsen et al., 2012; Bais et al., 2006). Additionally, genera such as *Gibberella* and *Paraphoma*, which are often reported as soil-borne or potential plant pathogens, were also detected in CS and RS, however, no symptoms in alfalfa roots were observed in this study. Microorganisms typically occupy multiple niches and can be isolated from diverse environments, with soil acting as a source library for microorganisms (Gibbons and Gilbert, 2015; Trevors, 2010). Previous studies have shown that metabolites from *Gibberella* can promote the growth and development of plants (Keswani et al., 2022); however, whether such a relationship exists here warrants further verification.

### 4.3 The network structure and topological properties of three types of soil

Network analysis showed more complex interactions among microbial groups in NS and RS than in CS, indicating stronger biotic interactions that may drive processes such as nutrient cycling and pathogen suppression. These topological shifts suggest that alfalfa promotes a more interconnected rhizosphere community, fostering cooperative functions essential for restoration (Faust and Raes, 2012). However, compared to NS, the establishment of eutrophies in the soil microenvironment after alfalfa planting is more beneficial (Xu et al., 2022; Zhou et al., 2022). Based on these results, we hypothesize that the microbial community in CS is simpler than in NS because changes in the host may enrich or expel certain soil taxa, which is consistent with the high diversity found in NS and RS (Ma et al., 2016). Previous studies have shown that planting alfalfa alters the soil microbial community and promotes the enrichment of symbiotic vegetative microorganisms and biocontrol bacteria (Chamkhi et al., 2022). The positive or negative interactions of nodes in the network indicate niche overlap or competition and adaptation among genera (Faust and Raes, 2012; Gao et al., 2022). The bacterial community was dominated by *Actinobacteria* and *Gemmatimonadota*, harboring eutrophy-associated bacteria responsive to alfalfa root exudates (Rojas Barreto, 2021; Zhalnina et al., 2018), while the fungal community was dominated by *Ascomycota* and *Basidiomycota*, taxa involved in complex-polymer degradation (Mendes et al., 2013; Manici et al., 2024). It should be emphasized that our inferences are based on amplicon data that include some unclassified taxa and do not directly measure function; shotgun metagenomics or metatranscriptomics could provide deeper insight. Therefore, whether these network hubs represent different functional groups needs to be studied in detail through metagenomics and trait-based assays to classify and analyze genera or strains at the appropriate level. Additionally, the 2-year timeframe may not capture long-term dynamics. Future research should explore functional gene profiling and longer-term monitoring to validate these microbial contributions to soil restoration.

## 5 Conclusion

Alfalfa cultivation in alpine desert lands improved soil quality and dynamically reshaping microbial communities over time. Diversity initially declined but then homogenized progressively, with stronger restructuring in the rhizosphere. Community diversity was structured by soil type and rhizosphere effects, SOM and pH were the primary drivers of microbial shifts. Dominant and core taxa did not fully overlap; the bacterial and fungal cores comprised only a small subset of genera. Higher network complexity in NS and RS (vs. CS) indicates greater ecological adaptability of alfalfa rhizosphere microorganisms. These results provide microbial support for soil remediation in high-altitude sandy areas. Overall, alfalfa promotes microbial-mediated restoration by enhancing soil fertility through shifts in nutrient cycling and community interactions.

## Data Availability

The datasets presented in this study can be found in online repositories. The names of the repository/repositories and accession number(s) can be found in the article/Supplementary material.

## Footnotes

1
www.majorbio.com
