# Supplementary material for: Changes in soil microbial community structure during the transformation from native soil to alfalfa cultivation soil in the Kunlun Mountain sand area, Xinjiang, China
Source: Front Microbiol. 2025 Dec 17;16:1702974. doi: 10.3389/fmicb.2025.1702974 (PMC12754178; doi:10.3389/fmicb.2025.1702974)
Supplement: Supplementary file 1 [file Data_Sheet_1.docx]

Supplementary figures


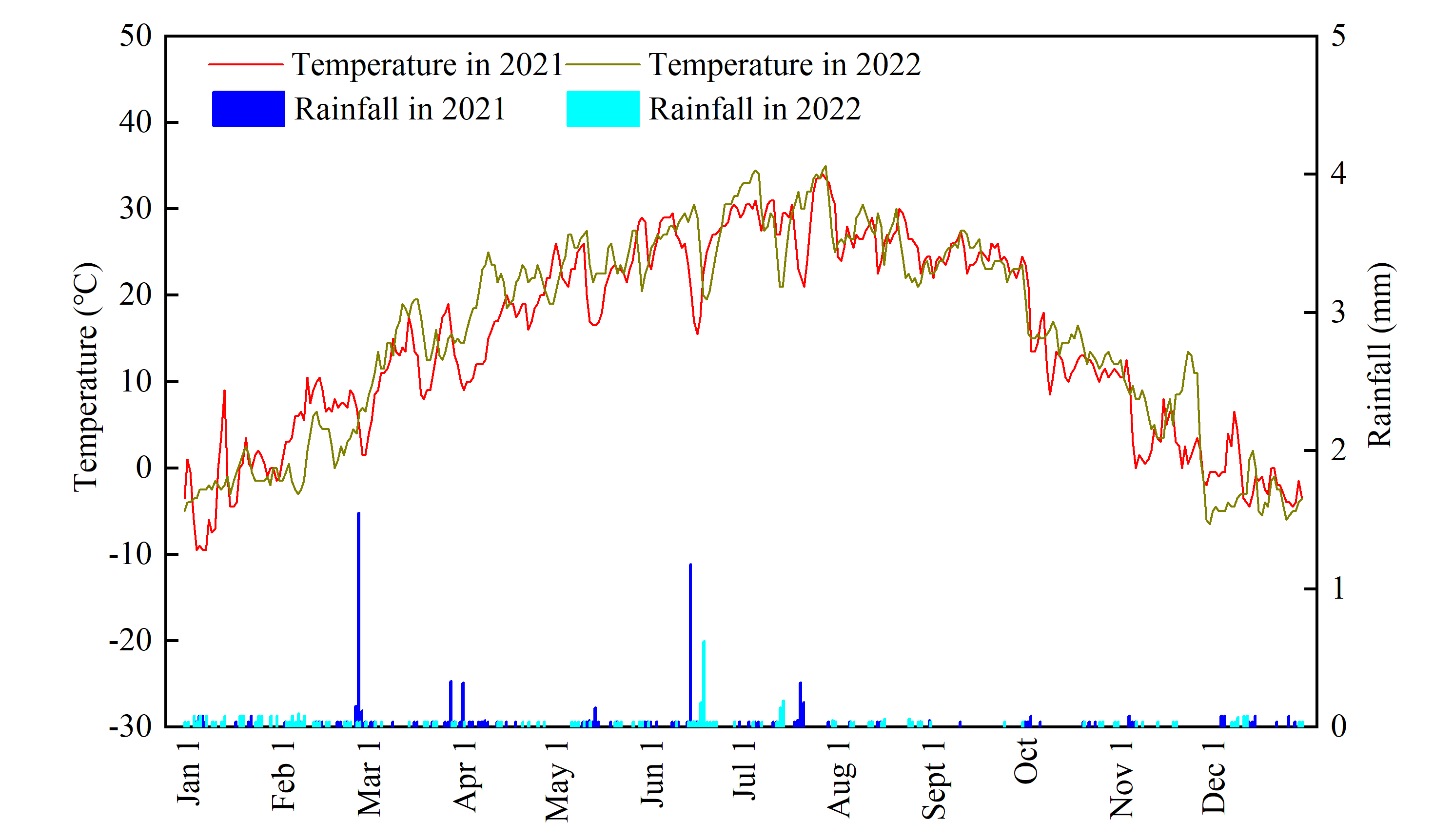


Figure S1. Temperature and precipitation in the study area in 2021 and 2022

The native land is a warm desert grassland, and the dominant species mainly include *Reaumuria songonica*, *Ceratoides latens*, *Stipa spp*., *Seriphidium spp*., etc., with a coverage of 8-10%. The average annual precipitation is 35.5 mm, the average evaporation is 2751.6 mm, the annual sunshine duration is 2690 h, the average annual temperature is 12.2 ℃, the extreme maximum temperature is 42.0 ℃, the extreme minimum temperature is -23.9 ℃, the average frost-free period is 235 days, the maximum wind direction is westerly, and the maximum wind speed is 28 m/s.

Figure S2. Rarefactions curves for chao1 and observed OTUs metrics.

Sparse curves are expressed by indices as follows: (a) Chao1 index of samples of estimated bacterial OTUs, (b) Chao1 index of soil types of estimated bacterial OTUs, (c) number of observed bacterial samples OTUs, (d) number of observed bacterial soil types OTUs; (e): estimated Chao1 index of samples of fungus OTUs, (f): estimated Chao1 index of soil types of fungus OTUs, (g): number of observed fungus samples OTUs, and (h): number of observed fungus soil types OTUs. NS, CS and RS represent native soil, alfalfa cultivated soil and alfalfa rhizosphere soil respectively. 21-1: first sampling (April 2021), 21-2: second sampling (October 2021), 22-1: third sampling (April 2022), 22-2: fourth sampling (October 2022).

Figure S3. Metric of microbial community structure in three types of soils.

Native soil (brown), cultivate soil (green) and rhizosphere soil (orange). In the picture, NS, CS and RS represent native soil, alfalfa cultivated soil and alfalfa rhizosphere soil respectively. 21-I: first sampling (April 2021), 21-II: second sampling (October 2021), 22-I: third sampling (April 2022), 22-II: fourth sampling (October 2022). a: PCoA only includes bacterial communities that grow in native soil samples and are sampled four times, b: cultivate soil, c: rhizosphere soil. d: PCoA only includes fungal communities that grow in native soil samples and are sampled four times, e: cultivate soil, f: rhizosphere soil. The reading of CSS conversion is used to calculate the Bray-Curtis distance in a, b, c, d, e and f.

Figure S4. Difference of bacteria OTUs in three types of soils.

A, B, C and D is the difference at the phylum level under four sampling times; and a, b, c, d is the difference at the genus level under four sampling times. Native soil (brown), cultivate soil (green) and rhizosphere soil (orange). Firmicutes increased significantly in rhizosphere soil at the 21-I, while Actinobacteriota did not change significantly. In the sampling of 21-II, the abundance of Proteobacteria in rhizosphere soil was significantly higher than that in cultivate and native soil, while the Chloroflexi and Acidobacteriota showed a transitional trend（Kruskal-Wallis test, *P*< 0.05, Falsely discovery rate corrected). At the genus level, 21-I *Exiguobacterium* was significantly enriched in rhizosphere soil, and *Nocardioides* was enriched from native soil to cultivate soil, but it did not appear in rhizosphere. *Microvirga* appears in cultivate and rhizosphere soil. In the follow-up sampling, the abundance of *Nocardioides*, *Skermanella* and *Microvirga* in cultivate and rhizosphere soil was significantly higher than that in the native soil (Kruskal-Wallis test, *P*< 0.05, Fdr-corrected).

Figure S5. Difference of fungal OTUs in three types of soils.

A, B, C and D is the difference at the phylum level under four sampling times; and a, b, c, d is the difference at the genus level under four sampling times. Native soil (brown), cultivate soil (green) and rhizosphere soil (orange). At the 21-I, the abundance of Ascomycota in native soil is higher than that in cultivate and rhizosphere soil, while Basidiomycetes are the opposite. while rhizosphere soil were enriched in some fungi of Basidiomycetes at the 21-I. At the 21-II, the cultivated soil and rhizosphere soil were enriched *Phaeomycocentrospora* and *Naganishia*. At the 22-I and 22-II showed that the rhizosphere soil was rich in *Naganishia*, and the cultivate soil was rich in *Gibberella*. *Talaromyces* were significantly enriched in cultivate soil and rhizosphere soil in the third sampling, but decreased significantly in the fourth sampling, and *Beauveria* was significantly enriched in rhizosphere soil.(Kruskal-Wallis test，*P*< 0.05，Fdr-corrected).


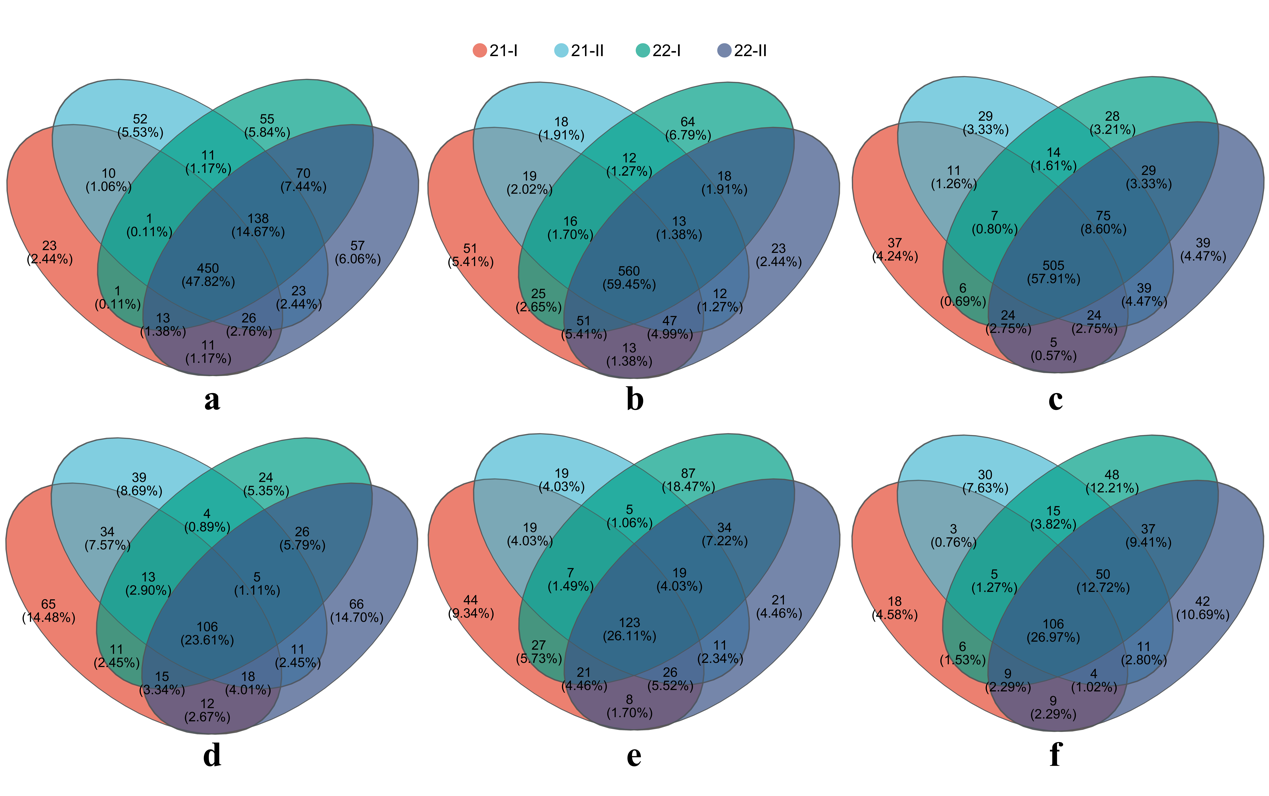


Figure S6. Endemic genera and common genera in three soils at different times.

(a): bacterial native soil, (b): bacterial cultivate soil, (c): bacterial rhizosphere soil, (d): fungal native soil, (e): fungal cultivate soil, (f): fungal rhizosphere soil. 21-I: first sampling (April 2021), 21-II: second sampling (October 2021), 22-I: third sampling (April 2022), 22-II: fourth sampling (October 2022).

Supplementary tables

Table S1 Physical and chemical characteristics of three soils in this study

| Item | Native soil | | | | Cultivate soil | | | | Rhizosphere soil | | | | *P*s | *P*t | *P*s×*P*t |
| --- | --- | --- | --- | --- | --- | --- | --- | --- | --- | --- | --- | --- | --- | --- | --- |
|  | NS 21-I | NS 21-II | NS 22-I | NS 22-II | CS 21-I | CS 21-II | CS 22-I | CS 22-II | RS 21-I | RS 21-II | RS 22-I | RS 22-II |  |  |  |
| SWC (%) | 8.75±0.15Cb | 8.40±0.22Bb | 5.35±0.27Cc | 9.88±0.11Ba | 10.52±0.39Bc | 12.89±0.32Ab | 6.91±0.25Bd | 15.67±0.29Aa | 12.20±0.39Ac | 13.61±0.32Ab | 9.90±0.36Ad | 16.19±0.56Aa | ** | ** | ** |
| pH | 7.06±0.06Ac | 7.10±0.06Abc | 7.24±0.03Aab | 7.31±0.02Aa | 6.92±0.04Ab | 6.97±0.05Ab | 7.15±0.02Aa | 6.99±0.04Bb | 6.76±0.05Bb | 6.97±0.07Aa | 6.90±0.05Bab | 6.93±0.08Ba | ** | ** | NS |
| BD (g/cm^3^) | 1.39±0.00Aa | 1.38±0.00Aa | 1.37±0.00Aa | 1.37±0.00Aa | 1.38±0.00Aa | 1.38±0.00Aa | 1.37±0.00Aa | 1.37±0.00Aa | 1.39±0.00Aa | 1.37±0.02Aab | 1.36±0.00Ab | 1.35±0.00Ab | ** | * | NS |
| EC (μS/cm) | 2.97±0.46Ba | 3.28±0.55Aa | 3.34±0.26Aa | 3.30±0.37Aa | 4.32±0.31Aa | 3.67±0.38Aab | 3.03±0.34Ab | 3.68±0.36Aab | 3.94±0.33ABa | 3.68±0.39Aa | 3.55±0.46Aa | 3.65±0.38Aa | NS | NS | NS |
| TN (g/kg) | 0.19±0.01Ca | 0.18±0.01Ca | 0.20±0.01Aa | 0.21±0.01Ba | 0.37±0.02Aa | 0.23±0.01Bb | 0.21±0.01Ab | 0.24±0.01Bb | 0.32±0.01Ba | 0.28±0.02Ab | 0.22±0.01Ac | 0.29±0.02Ab | ** | ** | ** |
| AN (mg/kg) | 31.80±0.96Ba | 30.86±0.98Ba | 31.69±0.67Aa | 30.70±0.96Aa | 36.37±0.83Aa | 34.73±0.92Aa | 31.62±0.50Ab | 31.88±0.20Ab | 37.31±0.71Aa | 37.03±1.11Aa | 33.75±0.77Ab | 32.76±0.82Ab | ** | ** | * |
| TP (g/kg) | 0.36±0.01Aab | 0.38±0.00Aa | 0.32±0.01Ac | 0.34±0.02Abc | 0.34±0.00Aa | 0.35±0.01ABa | 0.33±0.01Aab | 0.30±0.01Bb | 0.35±0.01Aa | 0.32±0.01Ba | 0.33±0.01Aa | 0.29±0.01Bb | ** | ** | * |
| AP (mg/kg) | 22.00±1.56Aa | 20.65±0.87Aa | 22.72±0.73Aa | 20.05±1.39Ba | 22.24±1.16Aa | 22.26±0.78Aa | 22.14±0.48Aa | 21.80±0.39ABa | 22.84±1.10Aa | 22.40±0.89Aa | 23.71±0.46Aa | 23.85±0.45Aa | NS | * | NS |
| TK (g/kg) | 3.88±0.08Aa | 3.94±0.04Aa | 3.94±0.13Aa | 3.95±0.13Aa | 3.73±0.03Aa | 3.86±0.06Aa | 3.97±0.06Aa | 3.92±0.07Aa | 3.90±0.05Aa | 3.94±0.08Aa | 3.94±0.12Aa | 3.95±0.09Aa | NS | NS | NS |
| AK (mg/kg) | 190.22±3.66Ba | 188.78±9.66Ba | 155.73±2.66Ab | 149.15±2.66Ab | 221.66±3.66Aa | 202.37±4.66Ab | 147.88±4.66Ac | 147.62±4.66Ac | 217.86±1.66Aa | 206.42±6.66Ab | 148.19±1.66Ac | 148.42±7.66Ac | ** | ** | ** |
| SOM (g/kg) | 3.86±0.12Ba | 4.12±0.13Ba | 3.94±0.13Ba | 3.80±0.23Ba | 6.65±0.43Ab | 7.86±0.16Aa | 5.65±0.26Ac | 6.62±0.30Ab | 6.66±0.26Ab | 7.98±0.19Aa | 5.74±0.29Ac | 6.61±0.33Ab | ** | ** | ** |
| MBC (mg/kg) | 86.27±1.05Ba | 86.71±1.51Ba | 78.72±2.24Ba | 78.86±1.92Ba | 89.25±2.04Bb | 102.16±2.92Aa | 83.89±4.88ABb | 83.68±4.10ABb | 112.56±4.44Aa | 109.03±2.41Aa | 90.43±2.53Ab | 90.50±2.53Ab | ** | ** | * |
| MBN (mg/kg) | 15.98±1.40Cb | 18.54±0.87Ca | 9.66±0.14Cc | 9.68±0.15Cc | 18.33±0.84Bb | 21.15±0.76Ba | 17.43±0.50Bb | 17.07±0.86Bb | 22.80±0.90Aa | 23.65±0.72Aa | 19.78±0.40Ab | 19.50±0.18Ab | ** | ** | ** |
| S_UE [mg/(g·d)] | 0.36±0.01Aa | 0.33±0.01Ba | 0.30±0.01Bb | 0.30±0.01Ab | 0.36±0.01Aa | 0.37±0.01Aa | 0.35±0.01Aa | 0.32±0.01Ab | 0.38±0.01Aab | 0.40±0.01Aa | 0.37±0.01Ab | 0.31±0.00Ac | ** | ** | * |
| S_NP [mL/(100g·2h)] | 0.32±0.02Aab | 0.33±0.01Aa | 0.32±0.01Ccb | 0.29±0.01Bb | 0.33±0.00Aa | 0.35±0.01Aa | 0.42±0.01Bb | 0.38±0.02Aa | 0.33±0.01Aa | 0.34±0.01Aa | 0.45±0.01Aa | 0.41±0.01Aa | ** | ** | ** |
| S_SC [mg/(g·d)] | 3.74±0.06Ba | 3.64±0.09Ba | 3.71±0.07Ba | 3.62±0.12Ba | 4.35±0.08Aa | 3.99±0.12Ab | 4.19±0.11Aab | 3.92±0.05Ab | 4.38±0.08Aa | 4.16±0.09Aab | 4.28±0.15Aab | 4.08±0.10Ab | ** | ** | NS |
| S_CAT [mL/(g·20min)] | 2.09±0.03Bb | 2.16±0.04Bab | 2.21±0.06Ba | 2.21±0.04Ba | 2.23±0.03Aa | 2.26±0.04Ba | 2.27±0.02ABa | 2.23±0.02Ba | 2.27±0.04Aa | 2.38±0.03Aa | 2.36±0.06Aa | 2.35±0.03Aa | NS | ** | NS |
| SQI | 0.38±0.11Ba | 0.37±0.14Ba | 0.26±0.14Bab | 0.16±0.07Bb | 0.63±0.17Aa | 0.69±0.18ABa | 0.47±0.18ABa | 0.45±0.11Aa | 0.67±0.14Aa | 0.59±0.31Aa | 0.61±0.09Aa | 0.48±0.09Aa | ** | ** | NS |

The average values of three soils and four times are shown, followed by the standard deviation of the average values. Statistical analysis was made for different time of each soil type. Variance analysis and Duncan (*P* < 0.05) test were were used. Different capital letters indicate that there are significant differences among different soils in the same period; Different lowercase letters indicate the same treatment, and there are significant differences in different periods. Principal component analysis was conducted on 16 soil indicators, and five principal components with characteristic values greater than 1 were selected, with a cumulative contribution rate of 79.307%. Finally, six indicators, SOM, TP, BD, S _ NP, EC and AP, are selected as MDS indicators. The correlation coefficient between each index is weak. MDS reduces data redundancy and can better replace the complete data set to evaluate the topsoil quality in the study area. Then, the weight of each index in MDS is calculated according to the common factor variance, and the soil quality index is calculated according to the membership degree and weight of each index. NS, CS and RS represent native soil, alfalfa cultivated soil and alfalfa rhizosphere soil respectively. 21-I: first sampling (April 2021), 21-II: second sampling (October 2021), 22-I: third sampling (April 2022), 22-II: fourth sampling (October 2022).

Table S2 Network characteristics of 16SrDNA fragments of three soil microorganisms

|  | Network properties | Native soil | Cultivate soil | Rhizosphere soil |
| --- | --- | --- | --- | --- |
| bacteria | Number of nodes1 | 112 | 95 | 144 |
|  | Number of edges2 | 919 | 409 | 1109 |
|  | Positive edges3 | 612 | 338 | 1007 |
|  | Negative edges4 | 307 | 71 | 102 |
|  | Avg. number of neighbors | 16.411 | 8.611 | 15.403 |
|  | Network diameter | 5 | 5 | 7 |
|  | Network density | 0.074 | 0.048 | 0.054 |
|  | Characteristic path length | 2.105 | 2.399 | 1.933 |
|  | Clustering coefficient | 0.247 | 0.225 | 0.292 |
| fungi | Number of nodes1 | 78 | 76 | 122 |
|  | Number of edges2 | 159 | 148 | 439 |
|  | Positive edges3 | 158 | 147 | 424 |
|  | Negative edges4 | 1 | 1 | 15 |
|  | Avg. number of neighbors | 4.077 | 3.895 | 7.197 |
|  | Network diameter | 4 | 8 | 6 |
|  | Network density | 0.026 | 0.026 | 0.03 |
|  | Characteristic path length | 1.576 | 2.823 | 2.215 |
|  | Clustering coefficient | 0.235 | 0.244 | 0.225 |

1 Bacterial taxa (genus level) with at least one significant (*P* < 0.01) and strong (Spearman > 0.8 or <-0.8) correlation.

2 Number of connections/correlations obtained by Spearman analysis;

3 SparCC positive correlation (> 0.85 with P < 0.01); 4 SparCC negative correlation (< -0.85 with *P* < 0.01)
